# Longitudinal, genome-scale analysis of DNA methylation in twins from birth to 18 months of age reveals rapid epigenetic change in early life and pair-specific effects of discordance

**DOI:** 10.1186/gb-2013-14-5-r42

**Published:** 2013-05-22

**Authors:** David Martino, Yuk Jin Loke, Lavinia Gordon, Miina Ollikainen, Mark N Cruickshank, Richard Saffery, Jeffrey M Craig

**Affiliations:** 1 Cancer, Disease and Developmental Epigenetics, Murdoch Childrens Research Institute (MCRI), Flemington Road, Parkville, Victoria 3052, Australia; 2 Early Life Epigenetics Group, MCRI, Royal Children's Hospital, Flemington Road, Parkville, Victoria 3052, Australia; 3 Department of Paediatrics University of Melbourne, Royal Children's Hospital, Flemington Road, Parkville, Victoria 3052, Australia; 4 Bioinformatics Unit, MCRI, Royal Children's Hospital, Flemington Road, Parkville, Victoria 3052, Australia; 5 Hjelt Institute, Department of Public Health, University of Helsinki, Mannerheimintie 172, 00300 Helsinki, Finland; 6 Current address: Division of Leukaemia and Cancer Research, Telethon Institute for Child Health Research, Centre for Child Health, University of Western Australia, 100 Roberts Road, Subiaco, Western Australia 6008, Australia

## Abstract

**Background:**

The extent to which development- and age-associated epigenetic changes are influenced by genetic, environmental and stochastic factors remains to be discovered. Twins provide an ideal model with which to investigate these influences but previous cross-sectional twin studies provide contradictory evidence of within-pair epigenetic drift over time. Longitudinal twin studies can potentially address this discrepancy.

**Results:**

In a pilot, genome-scale study of DNA from buccal epithelium, a relatively homogeneous tissue, we show that one-third of the CpGs assayed show dynamic methylation between birth and 18 months. Although all classes of annotated genomic regions assessed show an increase in DNA methylation over time, probes located in intragenic regions, enhancers and low-density CpG promoters are significantly over-represented, while CpG islands and high-CpG density promoters are depleted among the most dynamic probes. Comparison of co-twins demonstrated that within-pair drift in DNA methylation in our cohort is specific to a subset of pairs, who show more differences at 18 months. The rest of the pairs show either minimal change in methylation discordance, or more similar, converging methylation profiles at 18 months. As with age-associated regions, sites that change in their level of within-pair discordance between birth and 18 months are enriched in genes involved in development, but the average magnitude of change is smaller than for longitudinal change.

**Conclusions:**

Our findings suggest that DNA methylation in buccal epithelium is influenced by non-shared stochastic and environmental factors that could reflect a degree of epigenetic plasticity within an otherwise constrained developmental program.

## Background

Epigenetic modifications such as DNA methylation play an important role in development, ageing and disease [1-3]. However, the factors that influence epigenetic dynamics are poorly understood. Twin studies have the potential to estimate genetic components of epigenetic state [4,5] and have demonstrated that gene expression and DNA methylation profiles can both be influenced by allelic, stochastic and environmental factors [6-10]. Non-shared environmental and stochastic factors together have been estimated to be the largest influence on promoter methylation *in utero *[7].

Studies of epigenetic change over time have predominantly used cross-sectional approaches and have focused on adults [11-17] or on intrauterine development [18,19]. A small number of such studies have assessed age-associated DNA methylation across wider time-spans, encompassing childhood, adolescence and adulthood [20-23]. Consistently, age-associated changes in DNA methylation are more likely to involve (1) increases in methylation; (2) genes associated with development, signaling and regulation of transcription; and (3) regions involved in epigenetic reprogramming during embryonic stem cell differentiation [12,14]. Since most of these studies have focused on CpG islands and promoter regions, age-associated epigenetic changes are incompletely characterized in relation to genomic coverage and life-course.

Longitudinal studies investigating aging and longevity have distinct advantages over cross-sectional designs, particularly in relation to controlling for genetic variation. For example, longitudinal studies directly query temporal sequences and pathways and individuals are studied rather than group averages. Longitudinal, array-based studies in blood from children in the first 1 to 5 years of postnatal life have shown similar results to adult studies with respect to gene function, genomic location and direction of age-related changes in DNA methylation, with the majority of age-related changes being observed in regions flanking CpG islands [24-26]. In adults, a high-resolution array-based study found a mixture of age-stable and age-dynamic variability throughout the methylome in adults [27]. Other studies of global DNA methylation in adults also showed a genetic influence on increase and decrease in DNA methylation [28] and a decrease in interspersed repeat DNA methylation over time [29].

A small number of epigenetic studies of ageing have focused specifically on twins. Cross-sectional studies have found that older monozygotic (MZ) twins differ more with respect to global and repetitive DNA methylation [30], a phenomenon referred to as 'epigenetic drift'. In contrast, using DNA from saliva, no evidence for such drift was found within MZ twins aged 21 to 55 years using a promoter/CpG island array [15]. A cross-sectional comparison of DNA methylation at the imprinted *IGF2/H19 *locus in adolescent and middle-aged MZ twins also found no evidence of epigenetic drift within pairs [31]. To our knowledge, only one study has examined DNA methylation in twins longitudinally, measuring three loci in buccal DNA from 46 MZ pairs and 45 dizygotic (DZ) pairs at 5 and 10 years of age [8]. This study revealed (1) locus-specific variability in DNA methylation; (2) change over time in individuals; (3) within-pair correlation and (4) epigenetic drift. Taken together, these studies show that epigenetic change over time is likely to be regulated by many factors, potentially in a tissue-specific and genome context-dependent manner. Longitudinal epigenetic studies in twins offer tremendous potential to further our understanding of the relationship between genetics and other factors that specify inter-individual temporal change in DNA methylation profile in humans.

We have used the Infinium HumanMethylation450 BeadChip (HM450) platform, which interrogates >485,000 CpG dinucleotides and contains probes from CpG islands, shores (2 kb regions flanking CpG islands), shelves (2 kb regions flanking shores), sites from 1,500 bp upstream of transcription start sites through to gene bodies and 3' UTRs, in addition to intergenic regions, regions involved in epigenetic reprogramming during embryonic stem cell differentiation and enhancers [32,33]. Although repeats are not covered by these arrays and intergenic regions are not covered to the same depth as genic regions, the platform represents a significant step towards genome-scale coverage. Using the Infinium HM450 platform, we have performed a longitudinal study of DNA methylation at birth and age 18 months in DNA from buccal swabs from 10 MZ and 5 DZ twin pairs from the Peri/postnatal Epigenetic Twins Study (PETS) cohort [34]. We report a large degree of epigenetic change during the first 18 months of postnatal life, with strong regional genomic biases for rate of change over time. We also present evidence for pair-specific levels of epigenetic change, suggesting a complex interplay between environment, non-shared environment and stochastic factors in molding the early postnatal epigenome.

## Results

### Data pre-processing

Our initial analysis of HM450 data included normalization of previously identified differences between Infinium I and Infinium II probes [35] using the SWAN method [36]. Stringent quality control steps to assess probe performance (see Materials and methods) and removal of all probes on × and Y chromosomes to minimize sex-specific effects, resulted in 53/60 samples (Table 1) with data from 330,168 probes remaining for downstream analysis.

**Table 1:** Twin pair characteristics

Twin pair ID number	Zygosity^a^	Chorionicity^b^	Twin 1 sex	Twin 2 sex	Gestational age	Birth weight discordance (%)^c^	Samples removed after QC^d^
1016	MZ	DC	M	M	37	5.4	T2_18
1022	MZ	MC	M	M	38	12.4	T1_18, T2 _18
1024	MZ	DC	F	F	37	12.1	T2_B
1032	DZ	DC	F	F	37	43.3	T1_18
1035^e^	MZ	MC	F	F	35	30.8	
1042	DZ	DC	M	M	30	19.8	
1046	MZ	DC	M	M	37	6.2	
1057	DZ	DC	M	M	37	13.3	
1058	MZ	DC	M	M	36	22.0	
1072	DZ	DC	F	F	37	14.5	
1107	MZ	MC	F	F	33	8.1	T1_18
1126	MZ	MC	F	F	32	27.3	T2_18
2034	MZ	DC	F	F	36	3.6	
3006	DZ	DC	M	M	37	3.6	
3014	MZ	MC	M	M	36	18.0	

^a^MZ, monozygotic; DZ, dizygotic. ^b^MC, monochorionic; DC, dichorionic. ^c^[(Weight of heaviest twin - Weight of lightest twin)/Weight of heaviest twin] × 100. ^d^T1/T2, twin 1/2; B, birth sample; 18, 18 months sample. ^e^Twin-to-twin transfusion syndrome.

### Determination of technical versus biological variation

**Figure 1:**
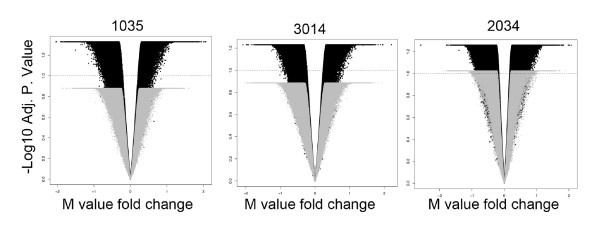
**Comparison of biological versus technical variation for matched replicate samples**. The data are represented as volcano plots of three MZ twin pairs (1035, 3014 and 2034; black) with an overlay of matched technical replicate DNA sample (gray). The x-axis represents the M-value fold change of variation across the four samples in each pair (replicate samples for each of twin 1 and 2 at birth and 18 months). The y-axis represents the -log10 FDR adjusted *P*-value for the moderated paired *t*-test. A genome-wide significance (FDR <0.1 for each individual pair) is denoted by the dotted horizontal line. In general, variation between biological replicates exceeds genome-wide significance and technical variation falls below genome-wide significance.

First to assess the sensitivity to detect biological variability between co-twins versus technical variation, we performed replicate hybridizations of three MZ twin pairs both at birth and 18 months. We compared the level of variation between co-twins (biological variation) to the level of variation between each technical replicate sample (technical variation). Biological variation (twin 1 versus twin 2) consistently exceeded technical variation (twin 1 versus twin 1; twin 2 versus twin 2) for each twin pair (Figure 1). We determined the average level of differential methylation between all biological and technical replicate arrays using a moderated paired *t*-test with false discovery rate correction. Precisely 230,340/330,155 probes were differentially methylated (adjusted *P*-value <0.05) across all biological replicates, whilst 858/330,155 probes were found to vary (adjusted *P*-value <0.05) across all technical replicates of twin pairs.

### Determining relationships between samples

Unsupervised hierarchical clustering of the entire dataset (Figure S1A in Additional file 1) revealed that most samples cluster according to age. The majority of co-twins also cluster together: 7/9 (78%) MZ co-twins cluster at birth and 6/6 (100%) at 18 months, while with DZ co-twins, 4/5 (80%) cluster at birth and 2/4 (50%) cluster at 18 months. To explore the variation in this dataset attributable to the effect of sequence variation on methylation values via *cis *genetic effects or probe hybridization, we performed hierarchical clustering selectively for probes overlapping known SNPs as defined by the HM450 SNP manifest (version 3, 103,148 probes). The results compared well to the full dataset: 7/9 MZ co-twins cluster at birth and 6/6 cluster at 18 months, while 4/5 DZ co-twins cluster at birth and 3/4 cluster at 18 months (Figure S1B in Additional file 1). Restricting this analysis to probes with reported SNPs at the CpG site assayed by the probe (2,527 probes in this data set) resulted in 8/9 MZ co-twins clustering at birth and 6/6 at 18 months (Figure S1C in Additional file 1). Interestingly, on average for this set of probes, DZ twins did not cluster with their co-twin; rather, DZ twins at birth clustered with their matched samples at 18 months. Thus, data for such probes are likely to reflect the genotype of the individual rather than representing purely methylation levels. A random sampling of the same number of SNP-associated probes did not reproduce this clustering (data not shown), indicating this effect did not represent a sampling bias. These results suggest that SNP-containing probes account for little variation in the overall data set, with the exception of probes with SNPs at the CpG site assayed.

### Identification of age-associated differentially methylated probes

**Figure 2:**
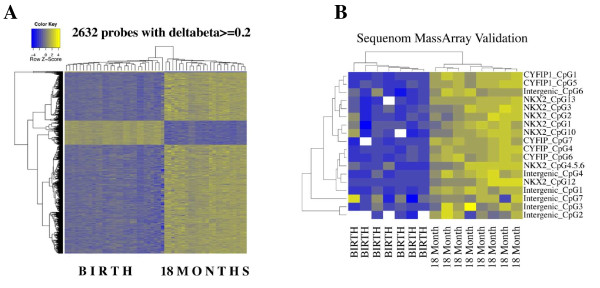
**Characterization of age-associated changes in DNA methylation**. **(a) **Heatmap of age-associated differentially methylated probes. Rows represent probes, columns represent samples. Cells are colored according to level of methylation (blue, hypomethylated; yellow, hypermethylated). Most age-associated changes involve an increase in methylation. **(b) **Heatmap of Sequenom EpiTyper validation data. Rows represent assayed CpG sites, columns represent samples. Cells are colored as in (a). Increases in methylation with age mirror those shown in (a).

**Figure 3:**
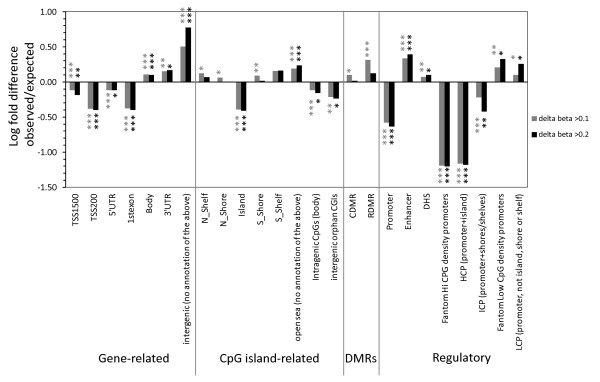
**Enrichment of aDMPs by genomic location**. Log-fold difference of enrichment (observed/expected frequency) in the aDMPs with *P *< 0.05 and delta beta >0.1 (*n *= 14,629) and >0.2 (*n *= 2,632) for specific genomic locations, grouped by association with genes, CpG islands, known DMRs and regulatory regions. Positive values indicate enrichment and negative values indicate depletion in the aDMP dataset. *P*-values: *P < 0.05; ** *P *< 1 × 10^-20^, *** *P *< 1 × 10^-50^.

To identify specific sites of differential DNA methylation associated with age, we used an empirical Bayes method [37] to compare birth samples with matched 18-month samples in all individuals and performed a probe-wise moderated paired *t*-test for differential methylation. Using this approach we found that 30.1% (99,198) probes changed significantly over time (adjusted *P*-value <0.05). These age-associated differentially methylated probes (aDMPs) changed by a mean β of 0.031 (3.1%) per year. Adding a further stringent cutoff of >20% absolute change over time to minimize technical effects [38] resulted in 0.8% (2,632) probes classified as stringent aDMPs (Table S1 in Additional file 2). Of these aDMPs, 87% showed a gain in DNA methylation over time whereas 13% showed reduced methylation (Figure 2a). We selected candidate aDMPs for validation based on their ranked change in methylation β value from birth to 18 months. The Sequenom MassArray Epityper platform was used to provide an independent measure of DNA methylation at aDMPs and confirmed the validity of the HM450 dataset. Using this approach, we confirmed that aDMPs identified by HM450 analysis are also representative of methylation at surrounding CpG sites (Figure 2b). Ontology and pathway analyses of the aDMP-associated genes showed an over-representation of cell development, morphogenesis (especially neuronal cells), and GTPase signaling pathways (Table 2; Tables S2 and S3 in Additional file 2). In order to determine whether aDMPs were more likely to occur at specific regions in the genome, we calculated the observed/expected frequency (enrichment) of genomic locations annotated in the HM450 manifest and assigned *P*-values with hypergeometric means tests. Intergenic regions were most likely to show changes in DNA methylation from birth to 18 months (Figure 3, grey bars; enrichment = 6.0×), followed by enhancers (2.5×) and 'open sea' regions >4 kb distant from CpG islands (1.7×). Promoters and CpG islands, but not their flanking shores and shelves, were less likely to show changed methylation over time (Figure 3; relative enrichment of 0.23× and 0.39×, respectively

**Table 2:** Ontology enrichment analysis of age-associated differentially methylated probes

GO term	Description	Adjusted *P*-value
GO:0000904	Cell morphogenesis involved in differentiation	0.0035
GO:0051056	Regulation of small GTPase-mediated signal transduction	0.0042
GO:0046578	Regulation of Ras protein signal transduction	0.0075
GO:0030182	Neuron differentiation	0.0157
GO:0031175	Neuron projection development	0.0192
GO:0048667	Cell morphogenesis involved in neuron development	0.022
GO: 0007409	Axonogenesis	0.026
GO:0048812	Neuron projection morphogenesis	0.027
GO:0000902	Cell morphogenesis	0.038

Gene Ontology (GO) terms significant at an adjusted *P*-value <0.05 [81] are shown. For full analysis, see Table S2 in Additional file 2.

### Identification of age-associated differentially methylated regions

**Figure 4:**
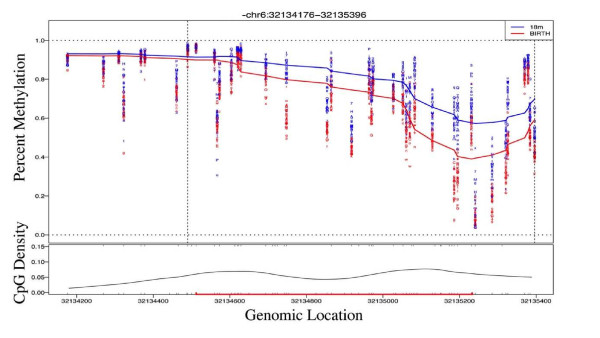
**Identification of age-associated differentially methylated regions**. Example of a DMR (*EGFL8*) identified by the peak-finding algorithm. The data show the loess-smoothed β values for all samples at birth (blue) and 18 months (red) according to genomic location. CpG density is shown below and a CpG island represented as a red line.

In order to identify larger regions of coordinated methylation change over time, we adopted a recently published differentially methylated region (DMR)-finding method [39]. This 'bump hunting' method identifies genomic regions in which clusters of consecutive CpG sites exhibit change over time in the same direction. Estimates were obtained for aDMRs by computing group medians and obtaining a value for the smoothed estimate that exceeds a t-statistic cutoff of 0.995. Using these criteria, we defined 897 aDMRs consisting of 4 or more consecutive probes changing in methylation between birth and 18 months (aDMPs). These aDMRs ranged in size from 33 to 1,698 bp. Twelve of these regions contained ten or more consecutive probes within approximately 1 kb of each other (Table S4 in Additional file 2 with an example shown in Figure 4). Of all aDMRs, 44% are located within 5 kb of a transcriptional start site, compared to 29% for aDMPs (Figure S2 in Additional file 1 and Table S4 in Additional file 2). Ontology analysis indicated that the aDMRs were significantly enriched for biological processes associated with cellular and organ development and in DNA binding (FDR <0.05; Table 3). As the sixth largest DMR (Table S4 in Additional file 2) and a representative of a number of the top age-associated ontologies, DNA methylation was validated at the cytoplasmic FMR1 interacting protein 1 (*CYFIP1*) gene in all samples using the Sequenom MassArray Epityper platform (Figure 2b).

**Table 3:** Ontology enrichment analysis for age-associated differentially methylated regions

GO term	Description	-log10 binomial *P*-value
GO:0048513	Organ development	17.68
GO:00048869	Cellular development process	15.59
GO:0030154	Cell differentiation	15.33
GO:0010033	Response to organic substance	14.47
GO:0009887	Organ morphogenesis	14.00
GO:0006359	Regulation of transcription from RNAs pol III promoter	9.66
GO:0000975	Regulatory region DNA binding	8.73
GO:0044212	Transcription regulatory region DNA binding	8.00
GO:0045945	Positive regulation of transcription from RNA pol III promoter	7.51
GO:0048598	Embryonic morphogenesis	6.80
GO:0016480	Negative regulation of transcription from RNA pol III promoter	6.67
GO:0003205	Cardiac chamber development	5.71
GO:0030326	Embryonic limb morphogenesis	4.4
GO:0060173	Limb development	3.82
GO:0035108	Limb morphogenesis	3.62
GO:0048546	Digestive tract morphogenesis	3.10

Gene Ontology (GO) terms significant at an adjusted *P*-value <0.05 [81] are shown.

### Epigenetic discordance within twin pairs at birth and 18 months

Within-pair epigenetic discordance resulting from non-shared environmental factors has been postulated to underscore variation in phenotypic traits [40,41]. We examined discordance in DNA methylation profile within twin pairs at birth and at 18 months of age. We calculated twin discordance as the absolute difference in β methylation values within pairs in birth samples and separately for 18-month samples. We ranked all probes according to average within-pair discordance at each age and performed 'ranked-list' ontology, which differs from 'gene-list' ontology in that there is no requirement for a predefined cutoff. All probes on the array were ranked by their scores for average within-pair discordance at each age (most discordant to least discordant), and the ranked list of probes was analyzed by the GOrilla bioinformatics tool [42] to identify ontology terms over-represented at the top of the list, compared with the bottom. We found that the most discordant genes at birth were consistently enriched for ontology terms associated with RNA metabolism, including spliceosome components and transcription factors (Table S5 in Additional file 2). At 18 months of age, the most discordant genes were associated with a similar set of gene ontologies as seen at birth (Table S6 in Additional file 2). The genes with discordant probes at both time points include a wide array of spliceosome components (for example, *WDR83 *and *CWC22*), zinc finger proteins (for example, *ZNF267*, *ZBTB1*, *ZNF10*), ribosomal proteins (for example, *RPS26*, *RPL15*, *RPL12*) and transcription factors (for example, *MAML1*, *HOXB13*).

We next investigated the distribution of DNA methylation discordance across genomic regions to determine whether discordance is more likely to occur at specific genomic locations. We have shown previously, using HM27 arrays, that median within-pair methylation discordance increased with increasing distance from CpG islands in three tissues (cord blood mononuclear cells, human umbilical vein endothelial cells and placenta) in both MZ and DZ twins at birth [7]. As the HM27 array focuses primarily on gene promoters and CpG islands, we repeated this analysis taking advantage of the diversity of genomic locations contained within the HM450 arrays. We calculated absolute within-pair discordance as before, and plotted probe discordance across genomic location at birth and at 18 months. The distribution of discordance values was consistent across all genomic annotations targeted on the array, with no evidence of regional enrichment (Figure S3A in Additional file 1). Similar results were observed selecting the top 10,000 most variable probes, or alternatively when the analysis was performed separately at birth and 18 months separately for both MZ and DZ twins (data not shown). We then filtered the dataset to include only probes present on the HM27 arrays and found evidence of higher levels of discordance around shores and shelves of CpG islands (Figure S3B in Additional file 1), which is consistent with our previously published observation with this platform [7].

### Level of change in epigenetic discordance (drift versus convergence) over the first 18 months is a pair-specific phenomenon

**Figure 5:**
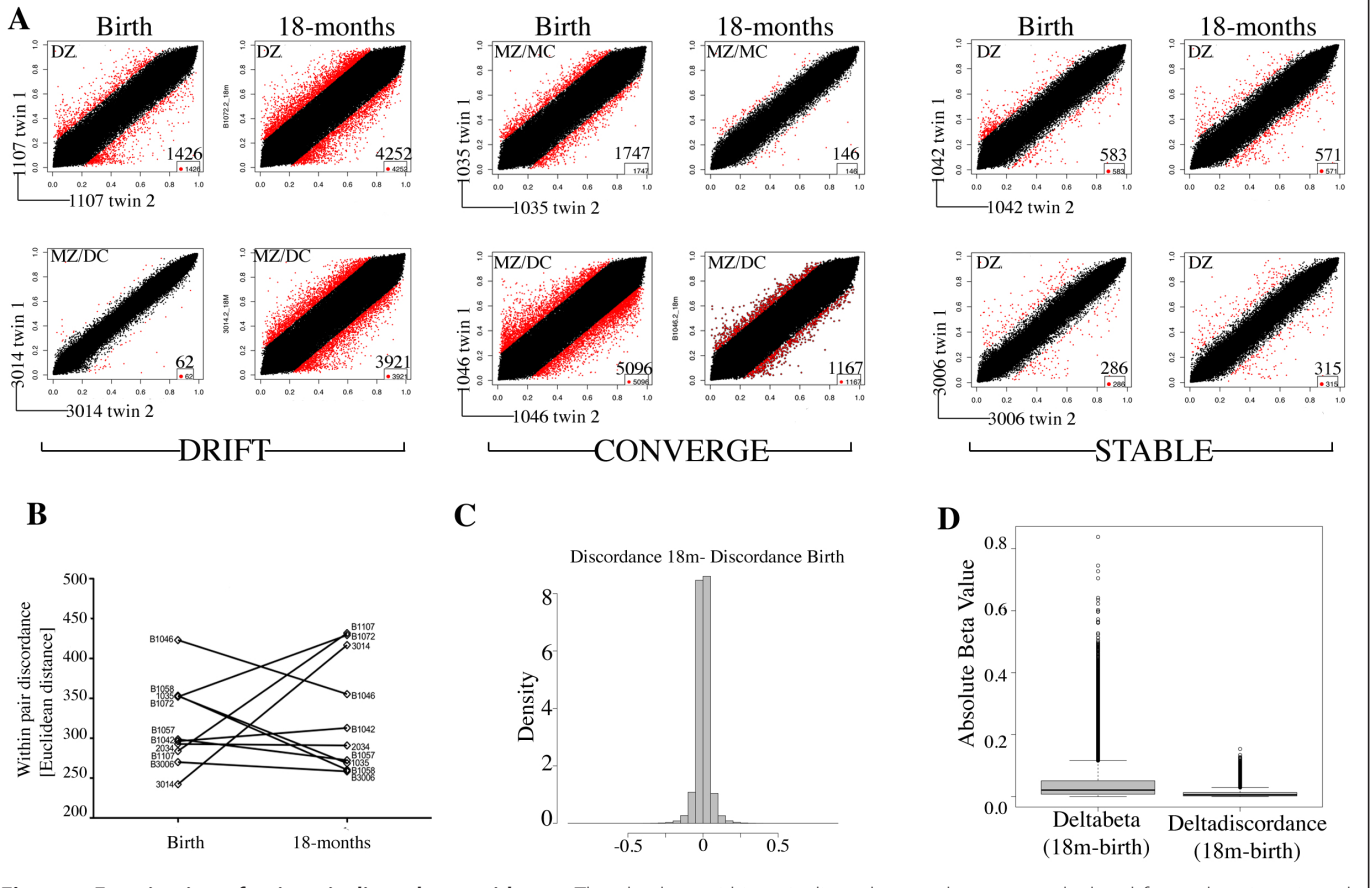
**Examination of twin-pair discordance with age**. The absolute within-pair discordance values were calculated for each twin pair and the change in discordance over time was assessed. **(a) **Scatterplots of methylation levels (β values) for six twin pairs versus their co-twin visualized at birth and at 18 months showing two examples each of pairs exhibiting within-pair drift, convergence and stability between birth and 18 months of age as defined in the text. Points shown in red represent those with an absolute within-pair discordance value of >0.2 (20%). The number of discordant probes is shown in the bottom right corner of each plot. MZ, monozygotic; DZ, dizygotic; MC, monochorionic; DC, dichorionic. **(b) **Euclidean distance of within-pair discordances plotted for each twin pair at birth and 18 months. Within-pair discordance increases in those pairs that drift and decreases in those that converge; stable pairs remain with similar values. **(c) **Distribution of the delta discordance values defined as absolute discordance at 18 months minus absolute discordance at birth. **(d) **Boxplot comparison of the change in beta values with age (deltabeta) versus change in discordance values with age (deltadiscordance).

Since previous cross-sectional studies suggest that epigenetic discordance in twins increases with age [30], we next investigated the degree of epigenetic drift from birth to 18 months of age within our twin pairs. The probe-wise level of within-pair discordance for CpG sites exhibiting a β-discordance value of greater than 0.2 (>20% discordant) was visualized at each age on scatterplots (Figure 5a, points in red). In contrast to the anticipated drift associated with age, we observed that the degree of within-pair discordance over time varies in a pair-specific manner (Figure 5a), with some pairs becoming more discordant in 18 month samples compared to birth samples (that is, epigenetic drift), some pairs becoming less discordant in 18 month samples, which we termed 'convergence', and others similarly discordant at both ages ('stable'). This was supported by Euclidean distance measures of twin discordance [7] (Figure 5b). These phenomena were not associated with zygosity or chorionicity, nor influenced by the effects of probes targeting SNPs on the array (Figure S4 in Additional file 1). We further calculated the change in discordance with age (delta discordance), as the difference in twin discordance (absolute values) from birth to 18 months. The distribution of delta discordance values was strongly centered about zero, with no evidence for overall skewing with age (Figure 5c). A comparison of the magnitude of the absolute values of differences in within-pair discordance over time (delta discordance) compared to the absolute values for methylation change over time (18 months - birth) indicated that age-related changes are far greater on average than changes to within-pair discordance (Figure 5d).

We next sought to explore epigenetic drift and convergence in more detail. As there were no probes that showed consistent differences across all pairs, our aim was to determine whether we could identify any gene ontologies associated with probes that consistently 'drift', 'converge' or remain stable among our twins over time. To address this we grouped our twin pairs according to their observed temporal methylation discordance patterns ('drifting' or 'converging'), measured as values of change in discordance (delta discordance). Within both of these categories, we calculated the within pair delta discordance for each probe, and ranked all probes on the average delta discordance across pairs. We performed gene ontology analysis on the ranked lists for each 'drifting' and 'converging' category and found both were similarly enriched for genes involved in development and morphogenesis (Table 4).

**Table 4:** Ontology enrichment analysis for drifting and converging pairs

GO term	Description	Adjusted *P*-value
Drifting pairs		
GO:0032502	Developmental process	3.21E-12
GO:0048856	Anatomical structure development	8.34E-10
GO:0048869	Cellular developmental process	2.15E-09
GO:0048598	Embryonic morphogenesis	2.61E-07
GO:0009653	Anatomical structure morphogenesis	9.50E-07
GO:0007389	Pattern specification process	2.36E-06
GO:0050793	Regulation of developmental process	3.86E-06
GO:0007166	Cell surface receptor signaling pathway	5.33E-06
GO:2000026	Regulation of multicellular organismal development	1.06E-05
GO:0023051	Regulation of signaling	2.59E-05
		
Converging pairs		
GO:0048856	Anatomical structure development	7.43E-08
GO:0032502	Developmental process	1.01E-07
GO:0048598	Embryonic morphogenesis	2.14E-05
GO:0050793	Regulation of developmental process	2.99E-05
GO:0022610	Biological adhesion	3.66E-05
GO:0007155	Cell adhesion	3.05E-05
GO:0045595	Regulation of cell differentiation	7.62E-05
GO:0048869	Cellular developmental process	1.95E-04
GO:0023051	Regulation of signaling	3.07E-04
GO:2000026	Regulation of multicellular organismal development	3.66E-04

The top ten Gene Ontology (GO) terms ranked by FDR (q values) ranked by mean delta discordance across 'drifting' and 'converging' pairs.

## Discussion

This study, examining DNA methylation profiles in buccal cells of young twins, has further confirmed the highly dynamic nature of the human epigenome postnatally, in agreement with previous studies in other tissues [20,21,24-26,43]. Buccal cells represent a key bio-resource for age- and disease-associated epigenetic association studies [8,12,44,45]. From a previous study [46] and our own unpublished data, this sample type comprises >90% squamous epithelial cells with <10% blood cells. Moreover, we minimized cell heterogeneity due to immune reactions by not collecting from infants with mouth infections. Almost a third of all HM450 probes in our final dataset showed significantly changed DNA methylation levels at FDR <0.05. Furthermore, the average absolute methylation change in these aDMPs was 4.7% (3.2% per year over 18 months) and almost 3% of aDMPs exhibited an absolute methylation change of >20%. These changes are similar in magnitude to those seen in blood from birth to one year of age using HM27 arrays and FDR <0.05 (3.9% of probes with changes >20%; average change of 9.2% per year) [24] and in T cells from birth to one year of age using HM450 arrays and FDR <0.01 (2.7% of probes with changes >20%; average change of 14% per year) [25]. In addition, our findings are of similar magnitude to a cross-sectional study of DNA methylation in the prefrontal cortex from human cadavers using HM27 arrays [47]. In combination with other cross-sectional studies [20,22], these cumulative data support the idea that rate of change of DNA methylation in the genome in any one tissue is highest *in utero*, possibly reflecting extensive cellular differentiation during organogenesis, and then declines in childhood, with a further drop in adulthood [20,21,43].

In the current study, most (approximately 90%) age-associated changes involved an increase in DNA methylation over time. This agrees with previous longitudinal studies of early childhood [24-26] and cross-sectional studies of placenta throughout gestation [48], peripheral blood in children [20], peripheral blood in adults [12,13,16], buccal cells in adults [12] and in a comparison of differences in various tissues between fetal and adult tissues [22]. A recent study comparing the entire methylomes of a newborn and a centenarian using bisulfite sequencing [23] observed an age-associated decrease in methylation in most genomic regions, including interspersed repetitive DNA, intergenic and intragenic regions. Although additional studies are needed to reconcile these discrepancies, it is also likely that age-associated methylation changes are dependent on genomic and tissue context, on the methylation analysis platform used and on sample size.

We found that aDMRs are more likely to be in intergenic and intragenic regions (Figure 3). Such regions were also enriched in aDMRs identified in multiple tissues in rats [49] and mice [50]. The intergenic regions identified in our study are single copy and overlap with enhancers (Figure 3). Such regions undergo the most dynamic changes accompanying differentiation of pluripotent stem cells [51]. Almost a third of aDMPs identified, and almost a half of aDMRs, lay within 5 kb of transcriptional start sites (Figure S2 in Additional file 1), implicating such regions in the regulation of gene expression. The higher proportion of aDMRs (44%) compared to aDMPs (29%) around gene promoters most likely reflects the higher CpG density and co-methylation (locally correlated methylation) within these regions [52]. However, this could also be due at least in part to the relatively wider HM450 probe spacing in intergenic regions.

Ontology and pathways analysis showed that approximately three quarters of the genes associated with stringent aDMPs and all aDMRs are implicated in development and morphogenesis (Tables 2 and 3; Table S2 in Additional file 2). Signaling pathways, including those based on GTPase signal transduction, pathways intrinsic to development, were also enriched, as has been observed in other studies of age-associated changes in methylation [50,53]. A bias towards genes involved in development has been seen in cross-sectional [13,20,54,55] and longitudinal [25,27] studies of human aging and in a study of embryonic stem cell differentiation [53].

It is interesting to note that approximately half of the top ten aDMP ontologies related to neural development (Table 2), a finding shared with previous studies of methylation in saliva [15] and blood cell fractions and buccal epithelium from adults [12] and in a large meta-analysis of multiple HM27 datasets from human brain and blood [55]. Further studies are needed to ascertain whether these findings relate to biases related to the large proportion of the genome expressed in the brain.

We found that despite a trend towards increased methylation with age in all regions of the genome, CpG-dense promoter regions were particularly depleted in aDMRs and CpG-poor promoters were moderately increased (Figure 3), contrary to previous cross-sectional [13,15,16,56] and longitudinal [26] studies of ageing-associated methylation change. This disagreement is most likely because HM27 arrays are enriched in CpG-dense CpG islands. However, our data agree with findings from studies using methods that include intergenic regions, that low CpG density promoters are enriched in mouse aDMRs [50], during differentiation of human embryonic stem cells [57] and between birth and very old age [23].

### Epigenetic discordance at birth and 18 months of age

Very few genome-wide studies of methylation or expression have been performed on buccal cells. One such study, of buccal cells collected from 20 twins aged 13 to 14 years using a low resolution CpG island array, found no significant methylation differences within pairs [44]. However, a study of smoking-induced differential gene expression in buccal cells identified a differentially expressed network of genes with, at the hub, transcription factors REL and CREB [58], which are among the top 10% most discordant genes at birth and 18 months in our data (Tables S5 and S6 in Additional file 2). Despite the extensive longitudinal changes in DNA methylation described above, we found that, in general, probes located within genes associated with RNA metabolism (for example, spliceosome components) and control of gene expression (for example, transcription factors) were consistently more discordant within twin pairs at both birth and 18 months of age. Of interest, this class of genes has previously been shown to have altered levels of transcription in buccal cancer [59].

### Epigenetic drift and convergence

In the current study, we found that a summed value (Euclidean distance) of epigenetic discordance across hundreds of thousands of loci can vary between and within pairs and can increase or decrease over time. In accordance with our genome-scale findings, a longitudinal study of DNA methylation at seven imprinted gene loci in buccal cells between birth and one year of age showed that inter-individual variation similarly increased, decreased or remained similar in singletons and that the direction of change could differ between individuals [45]. A longitudinal study of DNA methylation at three genes in buccal cells in 46 MZ and 45 DZ twin pairs found that methylation drifted in some pairs and converged in others over time [8]. Similar results were found for MZ and DZ twins and a role for genetic, shared and non-shared environmental factors, dependent on genomic location, in these longitudinal changes was postulated [8]. For MZ pairs, changes in within-pair discordance must be influenced solely by stochastic and non-shared environmental factors. Evidence for the latter comes from our previous studies of methylation in newborn twins [6,7,60] and from a cross-sectional study of DNA methylation in seven genes in whole blood from >200 MZ twin pairs aged 18 to 89 years [61]. Data from a longitudinal, genome-scale study of DNA methylation (using HM450 arrays) in whole blood from an independent cohort of young adults (aged 22 to 32 years) also provides evidence of genome-scale methylation drift and convergence defined by changes in Euclidean distance over time (Figure S5 in Additional file 1).

Epigenetic drift has been postulated to arise from the cumulative effects of (non-shared) environment and stochastic events [30,62,63], the latter influenced by epigenetic events such as promoter occupancy by transcription factors [64] and by errors made during the maintenance of DNA methylation profile following DNA replication [30,63]. Recent studies suggest that epigenetic drift may also reflect differing rates of change of methylation among the population [65]. Furthermore, others have argued that epigenetic variability (or noise) is itself genetically programmed and has evolved to mediate some degree of plasticity (via canalization) [66]. In contrast, we suggest that 'convergence' may involve sites of methylation equalization between co-twins, possibly reflecting regression to the mean as a contributing factor. Regression to the mean is a phenomenon in which it is a statistical certainty that individual phenotypes, such as growth patterns [67], shift to the population mean over time [68]. This explains why twins with birth weight discordance become more similar over time [69] and can be understood in terms of twin-specific uterine-specific restrictions being replaced postnatally by a greater degree of shared environment [69-71]. Indeed, the twins in the current study had a median weight discordance [(Weight of the heavier twin - Weight of the lighter twin)/Weight of the heavier twin] of 13.3% at birth and 2.8% at 18 months. Although caution is needed with interpretations from a small sample size, we note that 'converging' pairs were more likely to start with a higher within-pair discordance (mean Euclidean distance = 375) than the drifting pairs (mean Euclidean distance = 295) (Figure 5b), although this difference did not reach significance (*P *= 0.11). Clearly, larger longitudinal twin-based studies are needed to further investigate factors contributing to epigenetic drifting and convergence over time.

## Conclusions

We have conducted the first longitudinal study of epigenetic change in buccal cells in twins from birth, using a validated, genome-scale methylation array. We have shown evidence that the epigenetic profile of both MZ and DZ twin pairs can exhibit epigenetic drift or convergence early in postnatal development. As genes involved in development exhibited the largest absolute changes in methylation over time and the largest, smaller-scale changes within twin pairs, we conclude that the epigenetically driven developmental program is influenced to some extent by stochastic and/or non-shared environmental factors. Thus, canalization may be influenced by such factors, in addition to genetic factors as suggested by Waddington [72,73].

## Materials and methods

### Subjects, tissues and DNA extraction

Sample collection from twins at the time of delivery was carried out with appropriate human ethics approval from the Royal Women's Hospital (project number 06/21), Mercy Hospital for Women (project number R06/30), and Monash Medical Centre (project number 06117C), Melbourne and the study was conducted according to the Declaration of Helsinki principles. The twin pairs chosen for methylation array analysis are shown in Table 1. The 10 MZ pairs and 5 DZ pairs shared a similar sex ratio, gestational age and birth weight to the full group of 250 pairs. Buccal cells were collected with Catch-all Sample Collection Swabs (EPICENTRE Biotechnologies, Madison, WI, USA) and were stored at -20°C until DNA extraction, which was performed as previously described [60].

### Infinium HumanMethylation450 BeadChip data acquisition and processing

DNA samples (1 μg) were bisulfite converted using the Methyl EasyXceed bisulfite modification kit (Human Genetic Signatures, North Ryde, Australia), according to the manufacturer's instructions. Conversion efficiency was assessed by bisulfite-specific PCR. DNA samples were hybridized to Illumina Infinium Human Methylation450 (HM450) BeadChip arrays according to the manufacturer's instructions. Raw intensity data (IDAT) files were imported into the R environment (version 2.14.1) [74] and processed using the *minfi *package [75]. All analyses were performed in R using packages available from the Bioconductor project [76]. Data quality was assessed in *minfi *using plots derived from various control probes on the array. Poor performing probes defined as those with an average detection *P*-value >0.001 in one or more samples were removed from the analysis (*n *= 132,113). Data from five samples with an average detection *P*-value >0.05 and with evidence of poor bisulfite conversion efficiency were removed completely. Probes on the × and Y chromosomes were also discarded from all samples. The resulting data were pre-processed using the Illumina method within *minfi *and subset-quantile within-array normalization was performed [36] for combined normalization of Infinium type I and type II probes. The log2 ratio of methylated probe intensity to unmethylated probe intensity were calculated in *minfi *and the resulting M-values [77,78] were quantile normalized between arrays using the limma package [79]. Sample quality was further assessed using hierarchical clustering plots available in *minfi *and *lumi *[77] packages. Following this, three additional samples were removed as outliers constituting a final data set of 330,168 probes and 53 samples.

### Statistical analysis

Exploratory analysis of sample relationships was performed using unsupervised hierarchical clustering analysis with the Euclidean distance and complete linkage algorithm, and dendrogram was created using gplots [80]. Differential methylation analysis was performed on M-values using the *limma *package using a cutoff of FDR-corrected *P*-values <0.05 [81] and delta beta values >0.2. To study discordance among co-twins at the probe-level, a linear model was fitted to the M-values with twin-pair as a predictive factor to model the twin relationship. The level of discordance among co-twins was interpreted as the residual measurement for each CpG from the model-fit. For enrichment analysis, gene sets were populated with probe IDs using the annotated regions provided in the Illumina HM450 manifest file (version 1.1). Annotations used were classified as gene-related (TSS1500 and TSS200, regions from -1500 to -200 and -200 to the transcriptional start site respectively, 5' UTRs, first exons, gene bodies, 3' UTR and intergenic (no gene annotation)); CpG island-related (islands (also split into intragenic and intergenic)), shores (0 to 2 kb flanking islands), shelves (2 to 4 kb flanking islands) and open sea (>4 kb from islands) [82]); DMRs (associated with cancer (CDMRs) and induced pluripotent stem cell reprogramming (RDMRs); [83] and regulatory regions (promoters, enhancers and DNAse hypersensitivity sites, likely to be a mixture of promoters and enhancers [84,85]). Boxplots were produced to graph each category by discordance score. The 'bump-hunting' methods described by Jaffe and colleagues [39] were implemented using the *charm *package available in Bioconductor [86]. We used the 'dmrFinder' algorithm without covariate adjustment, using the default SPAN settings and specifying a minimum four probes, and a t-statistic cutoff to identify probes as being in a DMR at 0.995. For gene ontologies the GOrilla bioinformatics tool [42] was used to perform ranked-list ontology using the entire array content ranked by scores for discordance. Gene-list ontology enrichment was performed on significant gene lists (FDR <0.05) using the DAVID bioinformatics tool under the default settings [87]. Pathway analysis data were analyzed through the use of Ingenuity Pathway Analysis (Ingenuity Systems, Redwood City, CA, USA). The analysis tool GREAT (Genomic Regions of Annotations Tool) [88] was used to analyze the functional significance of aDMRs using the single nearest gene association rule within a 100 kb window.

### Sequenom MassArray target validation

Target validation was performed using the Sequenom MassArray EpiTYPER (Sequenom, San Diego, CA, USA) performed as previously described [18,60]. Amplicons were designed using Sequenom EpiDesigner software. Primers are listed in Table S7 in Additional file 2. In brief, amplification was performed after bisulfite conversion of genomic DNA with the MethylEasy Xceed bisulphite conversion kit (Human Genetic Signatures, North Ryde, Australia). All PCR amplifications and downstream processing were carried out at least in duplicate and the mean methylation level at specific CpG sites determined. Raw data obtained from MassArray EpiTYPING were cleaned systematically using an R-script to remove samples that failed to generate data for more than 70% of CpG sites tested [60]. Also, technical replicates showing ≥10% absolute difference from the median value of the technical replicates were removed and only samples with at least two successful technical replicates were analyzed. Samples were compared across each analyzable CpG site in the amplicon, as well as the mean across the whole amplicon.

### Data availability

Array data described in this manuscript have been submitted to the Gene Expression Omnibus public repository and are freely available under the accession number GSE42700.

## Abbreviations

aDMP/R: age-associated differentially methylated probe/region; bp: base pair; DMR: differentially methylated region; DZ: dizygotic; FDR: false discovery rate; HM450: Infinium HumanMethylation 450 BeadChip array platform; MZ: monozygotic; RDMR: reprogramming differentially methylated probe/region; SNP: single nucleotide polymorphism; UTR: untranslated region.

## Competing interests

The authors declare that they have no competing interests.

## Authors' contributions

RS and JMC conceived the study. YJL performed the Sequenom validation. DM, LG, MNC and YJL analyzed the data. MO contributed the Finnish methylation dataset. JMC and DM wrote the first draft of the manuscript. All authors participated in discussions related to analysis and interpretation, have read and approved the final manuscript.

## Supplementary Material

Additional file 1 **Supplementary figures and legends**. Click here for file

Additional file 2 **Supplementary tables and legends**. Click here for file
