# Supplementary material for: Longitudinal, genome-scale analysis of DNA methylation in twins from birth to 18 months of age reveals rapid epigenetic change in early life and pair-specific effects of discordance
Source: Genome Biol. 2013 May 22;14(5):R42. doi: 10.1186/gb-2013-14-5-r42 (PMC4054827; doi:10.1186/gb-2013-14-5-r42)

Supplementary figure legends

Figure S1. Hierarchical cluster analysis of twin samples. Methylation data from all probes showed that most samples clustered by twin pair and by age. (A). M, male; F, female; small boxes indicate zygosity (DZ, open box; MZ, black box); large boxes indicate clustering of pairs together; asterisks indicate “orphan” twins whose co-twins failed QC analysis; B, birth samples; 18m, 18 month samples. (B) Hierarchical clustering on all probes containing potential SNPs. (C) Clustering on all probes containing SNPs at the assayed CpG site.

Figure S2. Location of aDMRs in comparison to aDMPs

Location of aDMPs and aDMRs in relation to distance from transcriptional start sites. TSS-proximal sites were defined by the GREAT bioinformatic analysis tool as <5kb from TSS and proportions of all probes are shown as a proportion of all probes within 500kb of the nearest gene.

Figure S3. Boxplots of average within-pair methylation discordance according to genomic location. The absolute values of twin discordance were binned into genomic categories according to the Illumina manifest annotation. The figure shows combined data for birth and 18 months. (A) The top panel shows the breakdown by all probes on the array and (B) the bottom panel shows only probes that were annotated as previously occurring on the HM27 array.

Figure S4. Pair-specific epigenetic drift is not entirely explained by SNPs. Scatterplots of six twin pairs versus their co-twin visualized at birth and at 18 months. Points shown in red represent those with an absolute within-pair discordance value of greater than 20%. The

points show in yellow are probes targeting known SNPs. The number of discordant probes is shown in the bottom right corner of each plot.

Figure S5. Longitudinal changes in within-pair discordance in blood samples from young adult twins. The absolute within-pair discordance was calculated for each twin pair and the change in discordance over time was assessed. Euclidean distance of within-pair discordances was plotted for each twin pair measured twice at least 1 year apart. The age range of the twin pairs is 22 to 32.

Figure S1. Hierarchical cluster analysis of twin samples

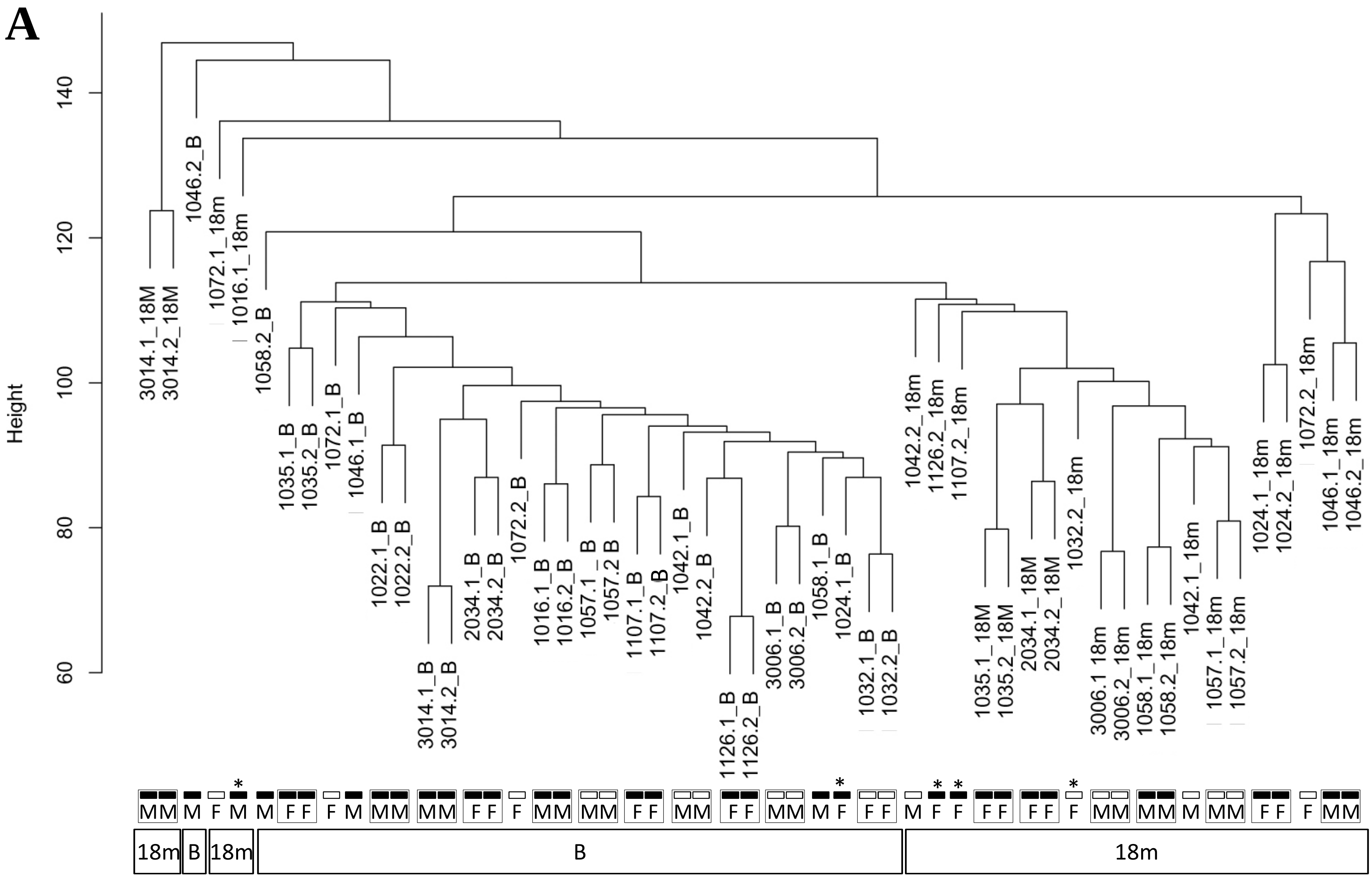

Figure S1. Hierarchical cluster analysis of twin samples

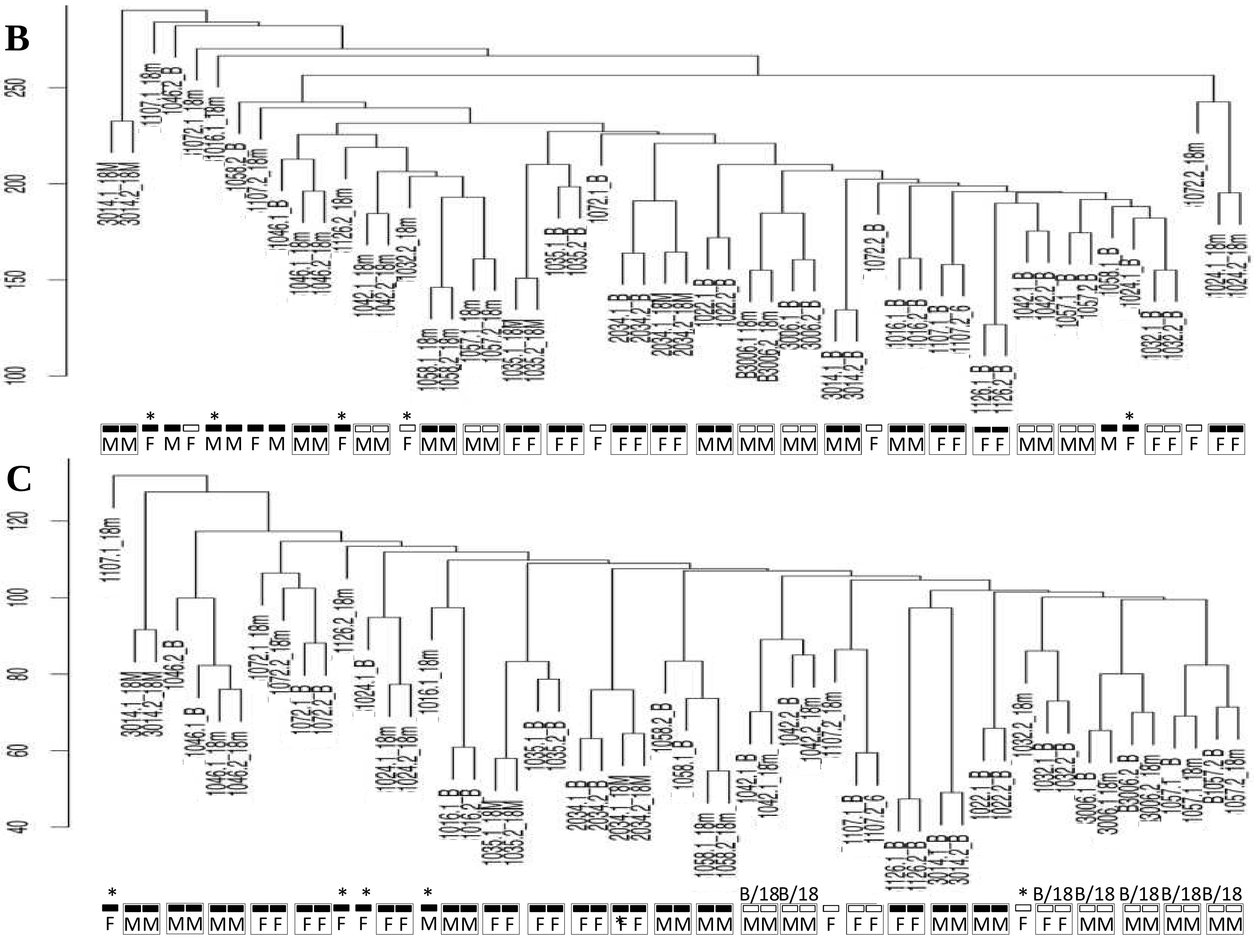

Figure S2. Location of aDMRs in comparison to aDMPs

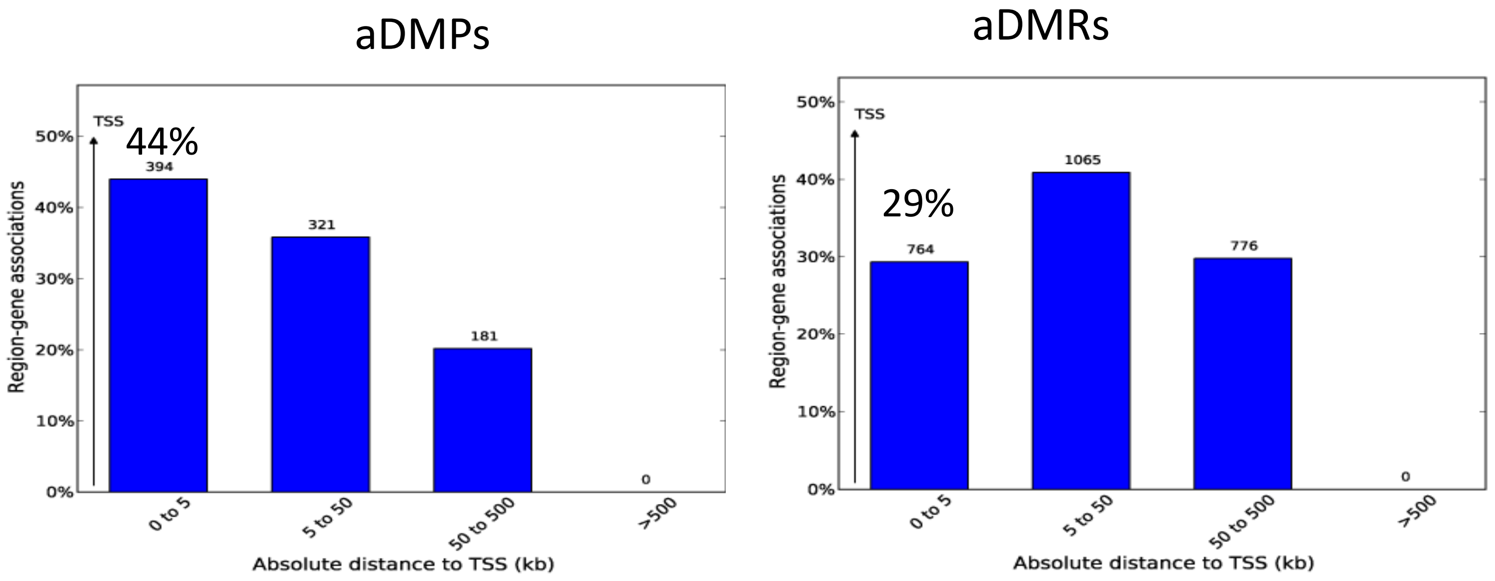

Figure S3. Boxplots of average-within pair methylation discordance according to genomic location.

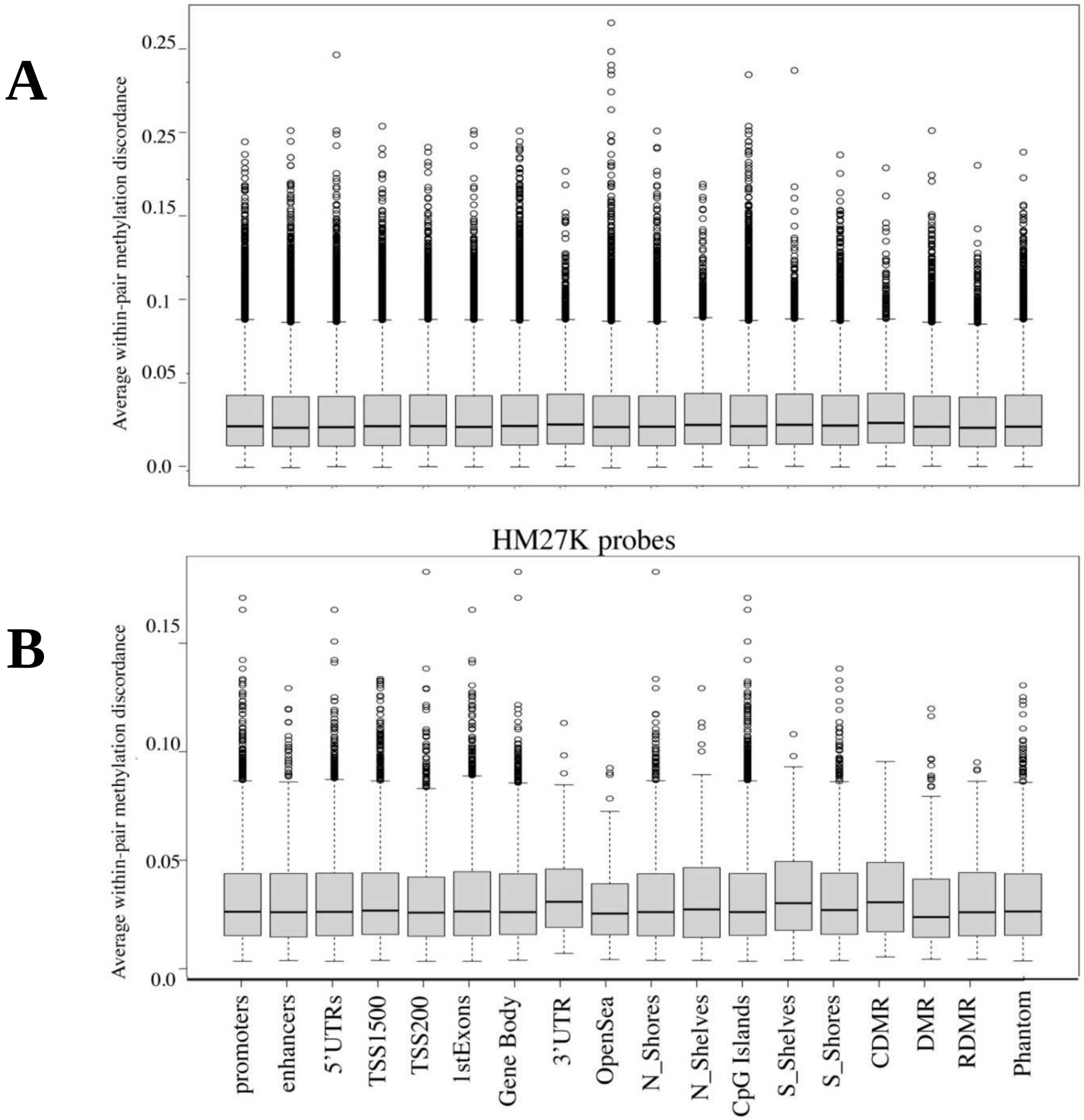

Figure S4. Pair-specific epigenetic drift is not entirely explained by SNPs

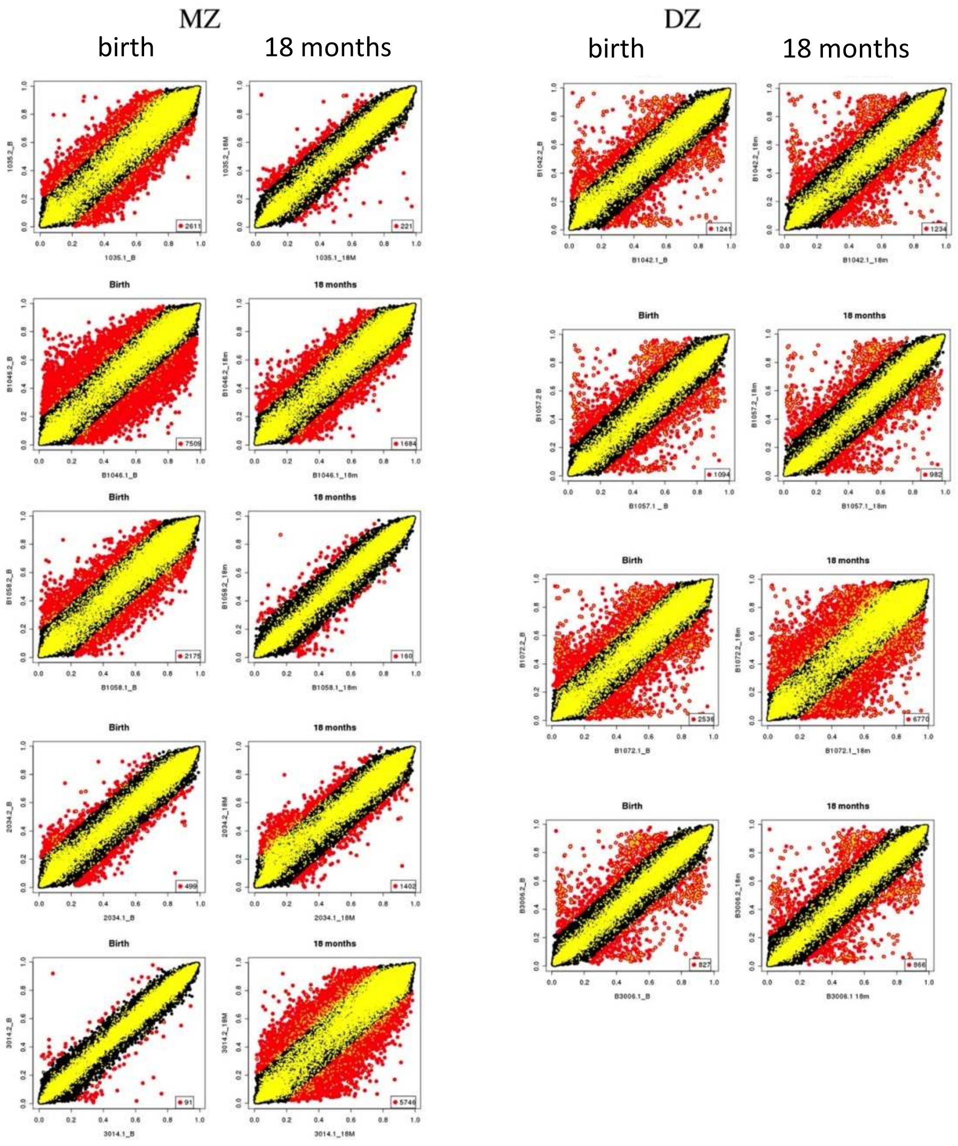

Figure S5. Longitudinal changes in within-pair discordance in blood samples from young adult twins.

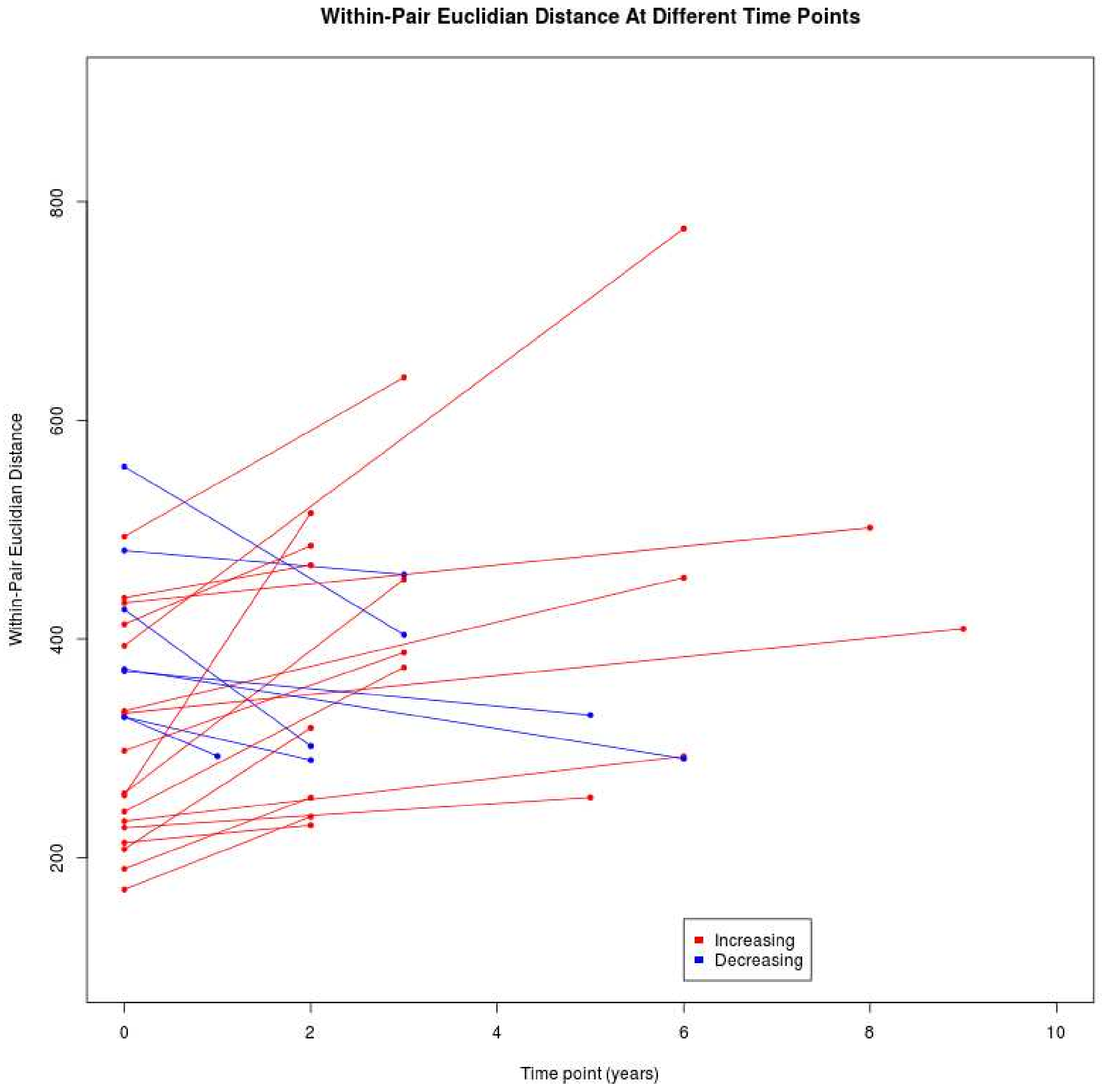

Supplement: Additional file 1 — Supplementary figures and legends. [file gb-2013-14-5-r42-S1.PDF]
